# Malaria hot spot along the foothills of Rakhine state, Myanmar: geospatial distribution of malaria cases in townships targeted for malaria elimination

**DOI:** 10.1186/s41182-019-0184-3

**Published:** 2019-12-18

**Authors:** San Kyawt Khine, Nang Thu Thu Kyaw, Pruthu Thekkur, Zaw Lin, Aung Thi

**Affiliations:** 1 Vector Borne Diseases Control Programme, Ministry of Health and Sports, Main Road, Sittwe, Rakhine State Myanmar; 2 International Union against Tuberculosis and Lung disease, Centre for Operational Research, Mandalay, Myanmar; 3 Centre for Operational Research, The Union South-East Asia Office, New Delhi, India; 4 Vector Borne Diseases Control Programme, Ministry of Health and Sports, Nay Pyi Taw, Myanmar

**Keywords:** Malaria elimination, Geospatial distribution, Rakhine State

## Abstract

**Background:**

Myanmar has targeted elimination of malaria by 2030. In three targeted townships of Rakhine state of Myanmar, a project is being piloted to eliminate malaria by 2025. The comprehensive case investigation (CCI) and geotagging of cases by health workers is a core activity under the project. However, the CCI data is not analyzed for obtaining information on geospatial distribution of cases and timeliness of diagnosis. In this regard, we aimed to depict geospatial distribution and assess the proportion with delayed diagnosis among diagnosed malaria cases residing in three targeted townships during April 2018 to March 2019.

**Methods:**

This was a cross sectional analysis of CCI data routinely collected by national malaria control programme. The geocode (latitude and longitude) of the address was analysed using Quantum Geographic Information System software to deduce spot maps and hotspots of cases. The EpiData analysis software was used to summarize the proportion with delay in diagnosis (diagnosed ≥24 hours after the fever onset).

**Results:**

Of the 171 malaria cases diagnosed during study period, the CCI was conducted in 157 (92%) cases. Of them, 127 (81%) cases reported delay in diagnosis, 138 (88%) cases were indigenous who got infection within the township and 13 (8%) were imported from outside the township. Malaria hotspots were found along the foothills with increase in cases during the rainy season. The indigenous cases were concentrated over the foothills in the northern and southern borders of Toungup township.

**Conclusion:**

In the targeted townships for malaria elimination, the high proportion of the cases was indigenous and clustered at the foothill areas during rainy season. The programme should strengthen case surveillance and healthcare services in the areas with aggregation of cases to eliminate the malaria in the township. As high majority of patients have delayed diagnosis, the reasons for delay has to be explored and corrective measures needs to be taken.

## Introduction

Globally, malaria is one of the top ten causes of death due to infectious diseases with an estimated 219 million malaria cases and 435,000 malaria deaths in 201 7[1]. It is estimated that nearly half of the world’s population is at risk of malaria.

Myanmar is one of the malaria endemic countries and accounts for almost 10% of all the malaria cases in South-East Asia Region (SEAR )[2]. Malaria control in Myanmar is complicated due to heterogeneity in distribution of disease and emergence drug resistant malaria. However, the country has made significant progress in reducing malaria incidence by 84% and mortality by 95% over 2012 to 2018. In spite of that, there were 76,518 malaria cases and 19 reported deaths due to malaria in 201 8[3]. Of all the malaria cases in 2018, *Plasmodium falciparum (Pf), Plasmodium vivax (Pv)* and mixed infection was seen in 50%, 47% and 3% cases respectivel y[3]. The National Strategic Plan (NSP) of National Malaria Control Programme (NMCP) of Myanmar aims to achieve malaria elimination by 2030 [4].

Rakhine state of Myanmar has high receptivity and vulnerability for malaria transmission with annual parasite incidence (API) of more than 1 per 1,000 population [5]. The NSP, targeted to reduce API <1/1,000 population by 2020 in the state. In 2018, the NMCP in collaboration with University of Research Co.,LLC (URC)-defeat malaria project, initiated a pilot project for elimination of malaria in three townships of Rakhine state. The project targeted to interrupt the transmission and achieve zero new indigenous malaria cases in these targeted townships by 2025 [6].

Three operational strategies were implemented to achieve elimination in targeted townships. 1) Improved access to malaria prevention, diagnosis and treatment through institution of community volunteers and project field staff. 2) Transformation of malaria surveillance into a core intervention through foci investigation and response, and 3) Comprehensive case investigation (CCI) with documentation of geolocation of patients’ residence and information on diagnosis and treatment of malaria cases [6].

Though the geolocation of malaria cases was collected during CCI, it is not been utilized under the project for visualizing the geographical distribution of malaria cases and identification of hotspots. The studies have reported that identifying and targeting hotspots is an effective and efficient intervention to reduce malaria transmission at all levels of transmission intensity [7–9]. In this regard, it is important to identify hotspots of indigenous malaria cases and target them for achieving the zero indigenous cases.

Similarly, the information obtained during CCI can help in monitoring the programme indicators like type of the case (indigenous, imported, etc.), timeliness of diagnosis and appropriateness of malaria treatment in the targeted townships. As such, the studies from Myanmar have had reported that only about 20% to 37% of malaria cases had early diagnosis (within 24 hours onset of symptoms [10–12]. Also, the proportion with early diagnosis varied across ethnicity, species of malaria, social support, availability of health personnel and distance from home to health facilities [13]. With improved access to diagnostic and treatment services, the targeted townships are supposed to have early diagnosis and adherence to treatment guidelines. However, there has been no such evaluation in these townships. The information on these indicators will help to set the feasible interim targets with this as a baseline measure.

In this regard, among malaria cases diagnosed during April 2018 to March 2019 and residing within the three townships targeted for malaria elimination in Rakhine state, we aimed to, a) describe the sociodemographic and clinical profile, b) to depict geospatial distribution and aggregation of malaria cases and c) determine the proportion with delayed diagnosis and associated factors.

## Methods

### Study Design

This was a cross sectional analytical study using secondary data routinely collected by NMCP.

### Study Setting

#### General setting

Myanmar is situated in Greater Mekong Sub-region with the population of 51.5 million and administratively divided into 14 states and regions and one Nay Pyi Taw Council territory [14]. Rakhine state with 17 townships is situated in the western part of Myanmar. The region has an average rainfall of 200 inches and has temperature range of 67-98'F which is conducive for breeding of mosquitoes and transmission of malaria. Rakhine state has highest number of malaria cases in Myanmar and contributes to about 20-25% of all cases in the country. During the past seven years period (2011-2017), there was a significant reduction of malaria morbidity and mortality. There was 87% reduction of malaria cases from 123,814 malaria cases in 2011 to 16,234 cases in 2017 [15].

**Fig. 1:**
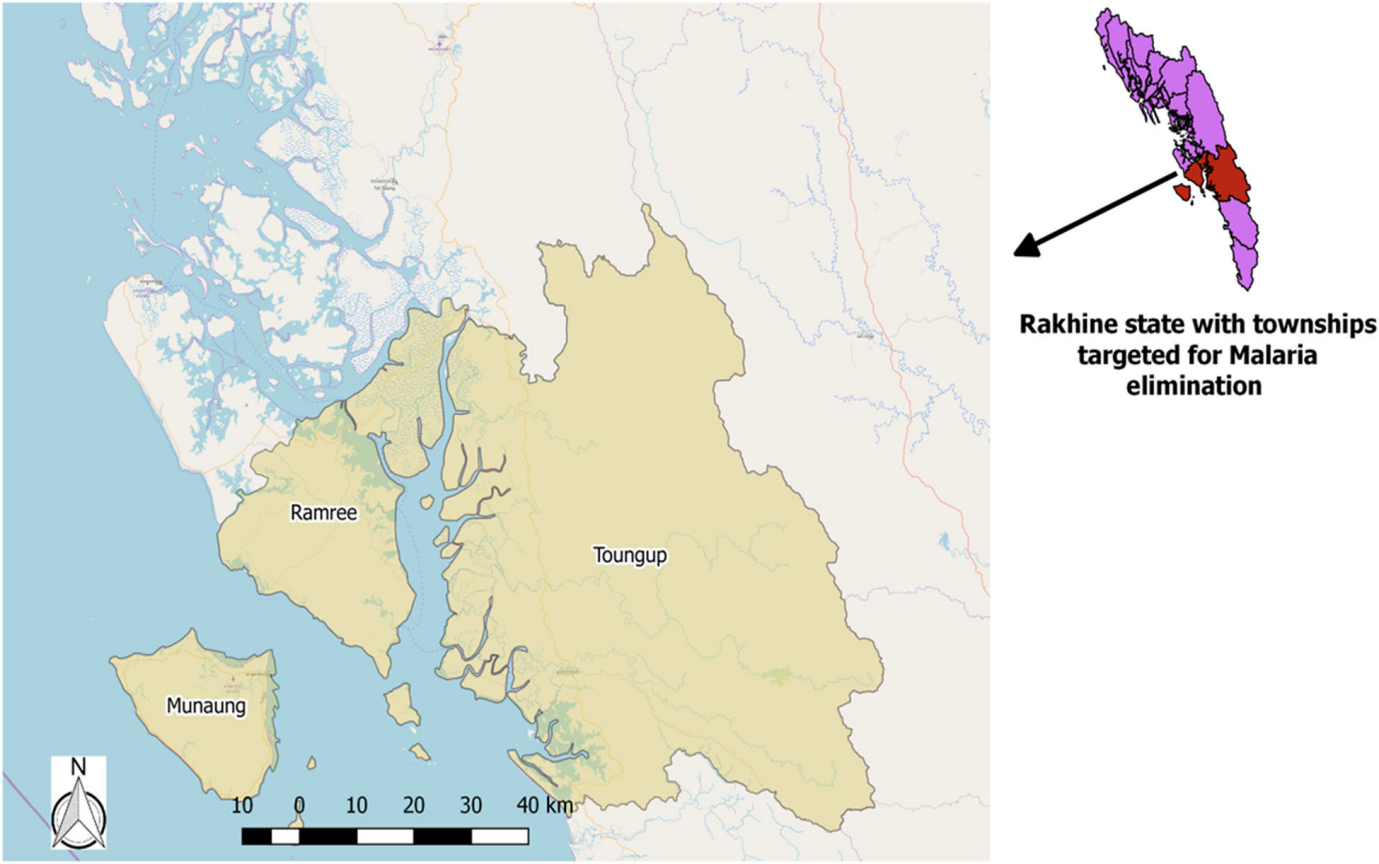
Map of Myanmar with study areas (townships targeted for malaria elimination in Rakhine State)

The Malaria Technical and Strategy Group (TSG) permitted to conduct elimination activities in three townships (Toungup, Ramree and Munaung) in Rakhine state in collaboration with University of Research Co.,LLC (URC)-defeat malaria project in 2018 [6]. The three townships are situated in the southern part of Rakhine state (Fig. 1) and about 300,000 individuals are living in these three townships. Annual parasite incidence in Toungup, Ramree and Munaung townships were 4.7/1,000 population, 0.3/1,000 population and no malaria cases in 2017 respectively [15]. The interventions under the project were started from April 2018 [6].

#### Specific setting

##### Malaria case diagnosis and treatment

Malaria case detection and management is provided at all the public health facilities and at village level by volunteers in Myanmar. All individuals presenting at health facilities or volunteers with fever are evaluated for malaria using either Rapid Diagnostic Test (RDT) or blood smear microscopy. The RDT used is a SD BIOLINE Malaria Ag Pf/Pv test. It detects histidine-rich protein II (HRP-II) antigen of Pf in human whole blood with sensitivity of 99.7%. Similarly, it detects plasmodium lactate dehydrogenase (pLDH) of Pv with sensitivity of 95.5%. The RDT has specificity of 99.5 % [16]. Blood smear microscopy is only available in hospitals.

Only those individuals tested positive for malaria with either RDT or blood smear are initiated on anti-malaria treatment. The patient details are recorded in the patients register. One copy of patient registers are reported to township Vector Borne Diseases Control (VBDC) programme at the end of the month. At township level, data enter to national malaria database (Access data) and send to state and central NMCP offices.

##### Comprehensive case investigation activity

In townships targeted for malaria elimination, all positive cases are notified to township investigation team within 24 hours of diagnosis to conduct case investigation. Township investigation team includes township malaria focal persons and health assistant from respective areas. Within three days, investigation team visits to the patient for case investigation using the standard form. In case investigation form, detail information of the patient, date of onset of fever, travel history before and during the fever to trace the source of infection and to consider onward transmission, blood transfusion history, past history of malaria and treatment taken, type of treatment give on this illness and type of the case (indigenous, imported, introduced, induced, relapse and recrudescent) are noted. The GPS co-ordinates of the house of the malaria patient is obtained using “Google Earth” mobile application and is noted on the case investigation form.

The team also conducts reactive case detection (testing malaria to family member and neighbor at the time of case investigation). All malaria cases are recorded in township malaria case register book after conducting the case investigation. The coordinator responsible for malaria elimination in the township enters the information in the case investigation forms into Microsoft Excel. The electronic data file is shared with state VBDC officials.

### Study population

All malaria cases diagnosed and residing in three townships targeted for elimination in Rakhine state during April 2018 to March 2019 were included in the study. Malaria case is defined as a person tested by RDT and/or microscopy and found positive and recorded in patient register regardless of the presence or absence of clinical symptoms [17]. The asymptomatic screening for malaria is done during response case detection among family member and neighbors of confirmed malaria case, individuals living in endemic areas, individuals with history of travel to malaria risk areas and individuals presenting with anemia or splenomegaly [18].

### Data variables, sources of data and data collection

The name, age, sex, address, date of diagnosis, type of diagnosis, type of malaria species, and type of provider of each malaria case were extracted from patient register database.

The name, age, sex, address, date of onset of fever, occupation (at risk/non-risk), residence area (rural or urban), travel history, past history of malaria, type of treatment given, bed net utilization (Yes/No), type of case (indigenous , imported or relapse defined according to WHO)[17,18]and GPS coordinates in degree decimal format (latitude, longitude) were extracted from the electronic case investigation database. The two excel database (patient register database and case investigation database) were merged matching “name” and “address”.

Areas with low population density and a land use which is predominantly agricultural were classified under ‘rural’ areas. In contrary, the areas which have an increased density of building structures, population and better infrastructural development were classified under ‘urban’ areas [14]. Cases with at risk occupation included forest workers, people worked in defense services and in development project in forest [4].

The geocode (latitude and longitude) of the address of the patient was obtained using Google Earth Pro version 7.3.2.5776 application. In cases where the CCI was not conducted, the village of the patient was used to geocode the address.

#### Data analysis and statistics

Data was analyzed using EpiData analysis software (version 2.2.2.182 EpiData Association Demark). Number and proportions were calculated to describe the socio-demographic, clinical and treatment profile of patients. Median (inter quartile range) day to diagnostic from onset of fever was calculated. Unadjusted prevalent ratios (PR) with 95% confidence intervals were calculated using log binomial regression to assess the socio-demographic and clinical characteristics associated with delayed diagnosis (>24 hours after onset of fever).

The Quantum Geographic Information System (QGIS) version 2.18.15 (QGIS Developer team, Las Palmas (2016)) was used to plot the malaria cases, and a map of geospatial distribution was constructed. The heat map depicting spatial heterogeneity was constructed with all the malaria cases diagnosed during study period. The Kernel shape quartic weight with raw values was used as a density measure [19, 20]. The fixed buffer radius of 2000 meters (the average flight range of the vectors – 2-3 km from their flight range was used for constructing heat map and hotspots [21].

The Fishnet layer (grids) with polygons of two kilometer length and breadth (4 square kilometer area) was created. The number of indigenous and imported cases of malaria within the each polygon was counted. The two grid maps, one each for indigenous and imported cases were created. In each of the created grid maps, the polygons with at least one malaria case were highlighted in the ‘red’ color.

The hot spots of malaria cases were created separately for summer, rainy and winter seasons. Initially, the heat maps were created for three different seasons with same conditions mentioned above. Each pixel of the generated heatmap had a ‘density value’ calculated using Kernel shape quartic weight. The median ‘density value’ across the pixels for summer season was used as a ‘threshold value’ (cut-off) for defining the hotspots. All the pixels with density value more than the ‘threshold value’ was considered as hotspot [22]. The same ‘threshold value’ was used for defining the hotspots for rainy and winter season to ensure comparability.

## Results

Of the total 175 malaria cases diagnosed in the study townships, the 171 (98%) cases resided within the townships were included in the study. The mean (standard deviation) age of cases was 26 (17) years, 122 (71%) were males, 163 (95%) were from Toungup township and 156 (91%) resided in the rural areas. Rapid diagnosis test (RDT) was used for diagnosis of malaria in 159 (93.0%) patients, 124 (73%) were diagnosed either by basic health staff or community volunteers and 134 (78%) had been infected with *Plasmodium falciparum*. The demographic and diagnosis related details of study participants is given in Table 1.

**Table 1:** Socio-demographic and clinical profile of malaria cases during April-2018 to March-2019, N=171

Characteristics	Frequency	(%)
Age in years		
Under five	14	(8.2)
5 to 14	39	(22.8)
15 to 24	38	(22.2)
25 to 34	33	(19.3)
35 to 44	18	(10.5)
≥ 45	29	(17.0)
Gender		
Male	122	(71.3)
Female	49	(28.7)
Area		
Urban	15	(8.8)
Rural	156	(91.2)
Township		
Toungup	163	(95.3)
Ramree	7	(4.1)
Munaung	1	(0.6)
Type of care provider		
Basic Health Staff	61	(35.7)
Volunteers	63	(36.8)
Doctors	33	(19.3)
Response team	14	(8.2)
Place of diagnosis		
Health centres	61	(35.7)
Hospitals	22	(12.9)
Clinics	11	(6.4)
Community	77	(45.0)
Diagnostic test used		
RDT	159	(93.0)
Blood Smear Microscopy	8	(4.7)
Both	4	(2.3)
Type of Malaria Species		
Plasmodium falciparum	134	(78.4)
Plasmodium vivax	35	(20.4)
Mixed	2	(1.2)

*Abbreviation: RDT* Rapid Diagnostic test,

**Fig. 2:**
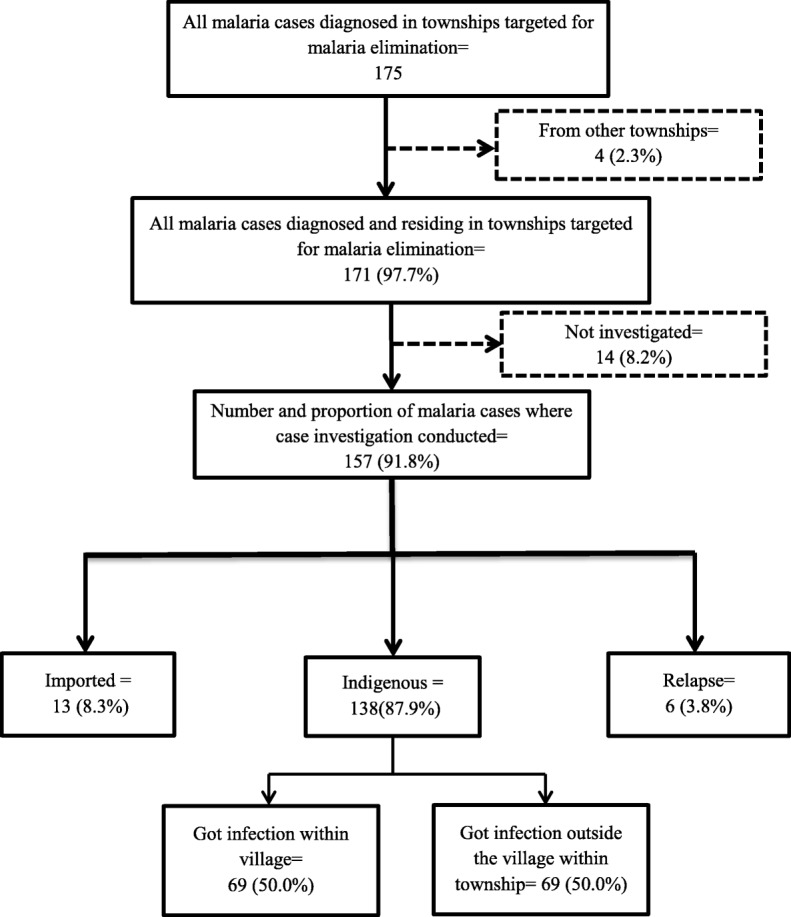
Flowchart on coverage of case investigation and delay time to diagnosis among diagnosed and resident malaria cases

The comprehensive case investigation (CCI) was conducted in 157 (92%) of the malaria cases and 138 (88%) cases were indigenous cases and 69 (50%) of indigenous cases got infection outside their village but within the township (Fig. 2). Among investigated cases, 63 (40%) had occupational risk, 38 (24%) had history of malaria in last 3 years, 78 (50%) travelled to malaria transmission area within one month before fever and 35 (22%) did not use bed nets. The median (inter quartile range) time to diagnosis was 3 (2-6) days from the onset of fever, 127 (81%) had delayed diagnosis and 136 (87%) received treatment according to national treatment guideline (NTG) (Table 2). The Table 3 shows the individual characteristics associated with delayed diagnosis. Males (PR-1.27 (95% CI-1.03-1.56)) compared to females, patients diagnosed at hospital (PR-1.29 (95% CI-1.09-1.53)) compared to those diagnosed in community and the indigenous but outside the village (PR-1.35 (95% CI-1.15-1.60)) compared to cases indigenous to villages had significantly higher risk of delayed diagnosis.

**Table 2:** Risk profile of investigated malaria cases during April-2018 to March-2019 (N= 157)

Characteristics	Frequency	Percentage
Occupation		
At-risk occupation	63	(40.1)
Non-at-risk occupation	94	(59.9)
History of Blood Transfusion		
Yes	2	(1.3)
No	155	(98.7)
History of malaria in last 3 years		
Yes	38	(24.2)
No	119	(75.8)
History of travel in past 30 days		
Yes, to transmission area	78	(49.7)
Yes, not to transmission area	19	(19.6)
No travel within 30 days	60	(38.2)
Type of Bed Net use		
Ordinary net	28	(17.8)
LLIN	94	(59.9)
Not used	35	(22.3)
Type of case		
Imported	13	(8.3)
Indigenous, source within village	69	(43.9)
Indigenous, source outside the village	69	(43.9)
Relapse	6	(3.8)
Delay from symptom to diagnosis		
≤24 hours	30	(19.1)
> 24 hours	127	(80.9)
Type of treatment		
According to NTG	136	(86.6)
Not according to NTG	21	(13.4)

*Abbreviation*: *LLIN* Long Lasting Insecticidal Net

*NTG* National Treatment Guideline , *P.f* Artemisinin based combination therapy (ACT ) three days and Primaquine stat dose : P.v or P.o- Cholorquine three days and Primaquine 14 days

Mixed- Artemisinin based combination therapy (ACT) three days and Primaquine 14 days: P.m- Chloroquine three days depending on age of patient

**Table 3:** Factors associated with delayed diagnosis (>24 hours of symptoms) among investigated malaria cases, N= 157

Characteristics	Total	Delayed Diagnosis, N	(%)	Unadjusted PR (95% CI)	p Value
Total	**157**	**127**	**80.1**		
Age in years					
< 5	12	9	75.0	Ref	
5 to 14	35	26	74.3	0.99 (0.68-1.45)	0.9609
15 to 24	33	25	75.8	1.01 (0.69-1.48)	0.9853
25 to 34	32	30	93.8	1.25 (0.89-1.75)	0.0809
35 to 44	17	14	82.4	1.10 (0.74-1.63)	0.6302
≥ 45	28	23	82.1	1.10 (0.76-1.58)	0.6049
Gender					
Female	47	32	25.2	Ref	
Male	110	95	74.8	1.27 (1.03-1.56)	0.0076
Occupation					
Non Risk occupation	94	76	59.8	Ref	
Risk occupation	63	51	40.2	1.00 (0.86-1.17)	0.9874
Type of Provider					
Volunteer	57	45	78.9	Ref	
Basic Health staff	56	48	85.7	0.92 (0.78-1.09)	0.3460
Doctor	32	28	87.5	0.90(0.75-1.09)	0.3133
Response team	12	6	50.0	0.63 (0.35-1.13)	0.0379
Place of diagnosis					
Community	69	51	73.9	Ref	
Health centres	56	48	85.7	1.16 (0.97-1.38)	0.1060
Hospitals	22	21	95.5	**1.29 (1.09-1.53)**	**0.0304**
Clinics	10	7	70	0.95 (0.62-1.45)	0.7935
History of malaria					
No	119	100	84.0	Ref	
Yes	38	27	71.1	0.85 (0.68-1.05)	0.0764
Type of the case					
Indigenous(within village)	70	49	70.0	Ref	
Indigenous(outside village)	81	75	93.6	**1.35 (1.15-1.56)**	**0.0002**
Imported	13	11	84.6	1.22(0.92-1.61)	0.2678
Relapse	6	3	50.0	0.71 (0.32-1.61)	0.3118

PR= Prevalence risk

*p* value <0.05 was considered significant can be added

**Fig. 3:**
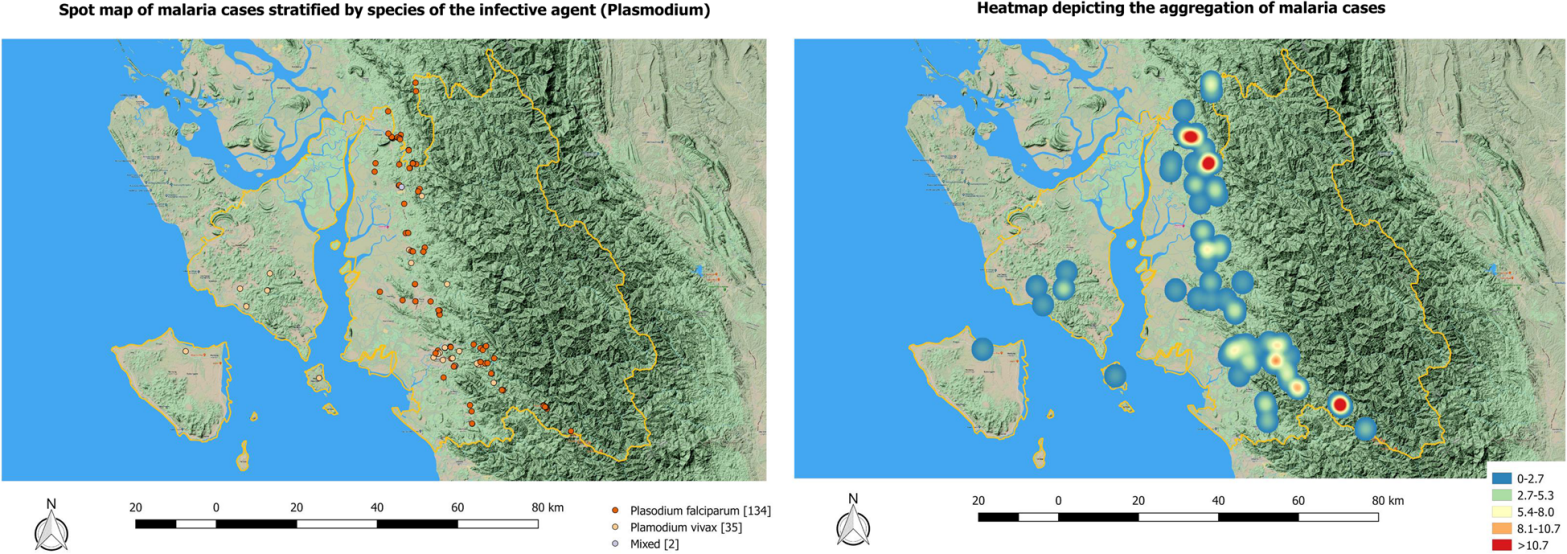
Spot map (stratified by type of species) and heat map of malaria

The Fig. 3 shows the spot map of all the malaria cases residing in the township, stratified by the type of species. There were no cases infected by *Plasmodium falciparum* in the Ramree and Munaung townships. In the Toungup township, the cases were distributed on the foothills. The heat map (Fig. 3) showed aggregation (red area) of cases on the foothills in northern and southern borders of the Toungup township.

**Fig. 4:**
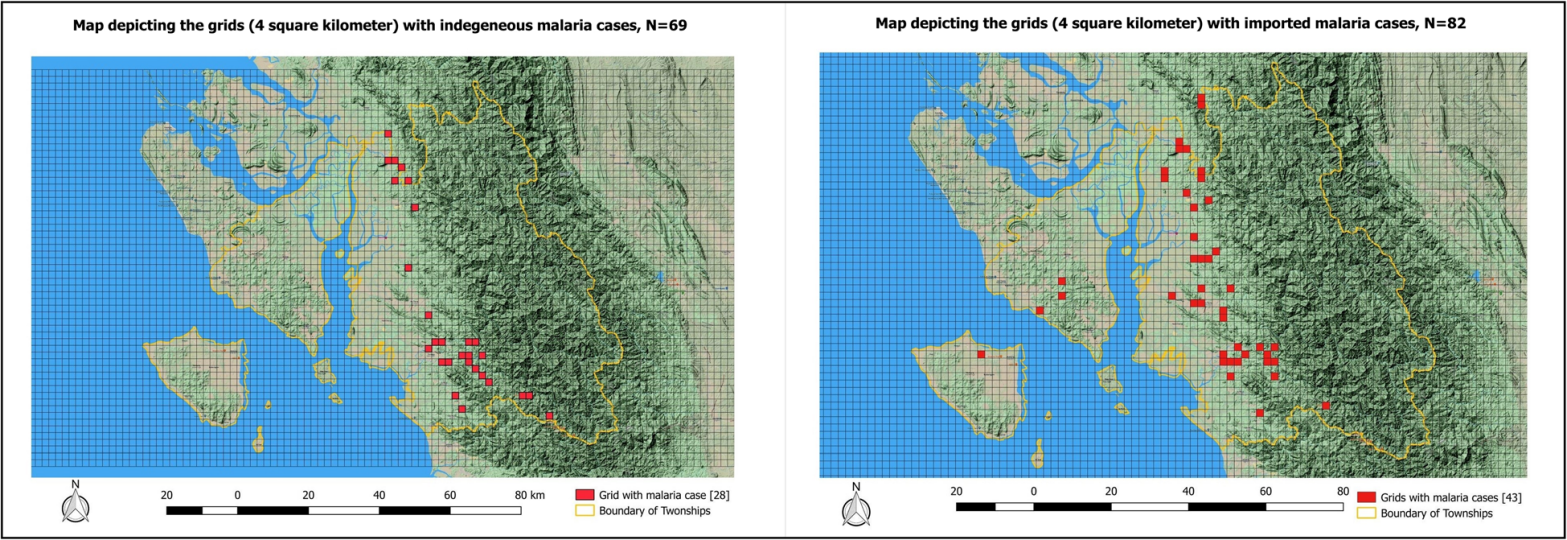
Maps depicting the equal size grids (4 square kilometre) with at least one indigenous (left) or imported (right) malaria cases

The Fig. 4 has two maps depicting the equal area grids (4 square kilometer) with at least one case indigenous to village or one case imported outside village or outside the township. The girds with indigenous cases within the villages were present only in the Toungup township and was limited over the foothills in northern and southern borders. The grids with cases imported outside the villages or outside the townships were present in all the townships and were not confined to specific region in Toungup township. There were 28 grids with indigenous cases and 43 grids with imported cases outside the village or township.

**Fig. 5:**
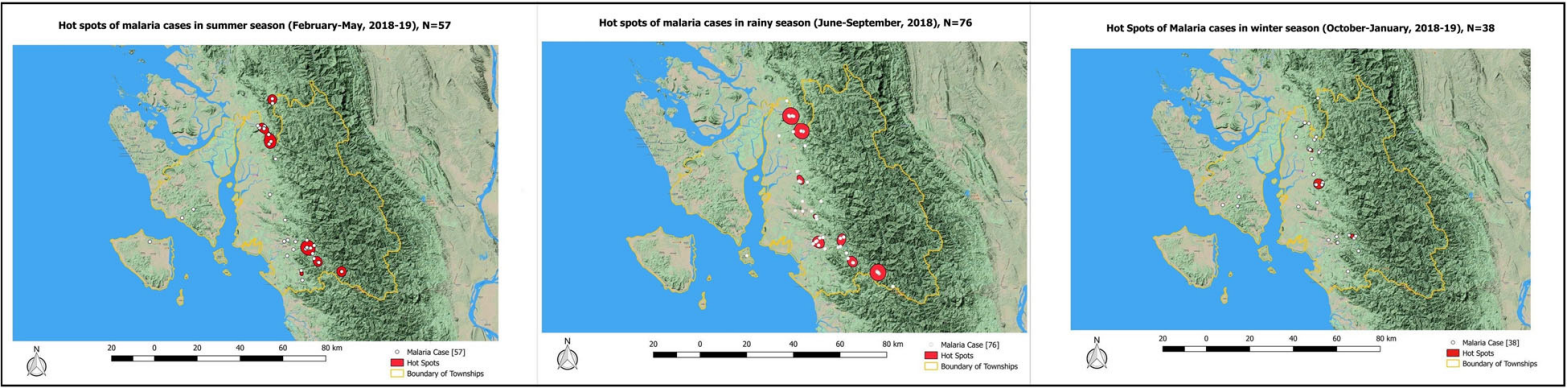
Hot spots of malaria cases during different seasons

The Fig. 5 shows the hotspots with heterogeneity of malaria case distribution in all the three seasons. The hotspots are located only in Toungup township across the three seasons. During the summer, the hotspots were located over the foothill in northern and southern borders of the township. During rainy season, the hotspots increased in size and spread across the foothill of the township. During the winter, small hotspot was located only over foothills in the central part of the township.

## Discussion

This is the first study in Myanmar using individual level clinical, demographic and geolocation data collected during CCI to assess the geospatial distribution and delayed diagnosis. Programmatically relevant important findings from this study are 1) there was seasonal and spatial heterogeneity with the malaria cases clustering during rainy season and at the foothill areas of Toungup township, 2) the cases those got infection outside the village or township were spread across the three study townships wherein the cases those got infection within the village were concentrated over the foothills of southern and northern borders of Toungup township, 3) more than 90% of the diagnosed malaria cases received CCI, 4) four in five malaria cases were delayed in diagnosis, 5) cases diagnosed at hospitals and cases imported outside the village within the township were found to be significant risk factors associated with delayed diagnosis.

There are a few strengths in this study. First, we obtained data of all diagnosed cases from both private and public sectors of the study townships and the case investigation information was available for more than 90% of the diagnosed cases. Thus, the study might not have been subjected to selection bias. Second, we geotagged the patient houses in more than 90% of the malaria cases. This prevented imprecision due to geocoding the villages or neighbourhoods and improved the validity of the heterogeneity maps. Third, we used routinely collected programme data which reflected the ground reality of the malaria control efforts in these townships.

There are some limitations in this study. First, the CCI coverage found in the study cannot be generalized to other townships of Myanmar. The study townships were better resourced as NMCP and URC-defeat malaria provided optimal number of staff to conduct the surveillance and response activities. Second, the village level shape files were not available for the study townships, thus we failed to calculate the rates of occurrence of malaria cases in each village. Third, we included malaria cases of only one year and thus, failed to assess the spatiotemporal variations in occurrence of the disease. Fourth, we failed to geotag or geocode the places where the imported cases probably acquired malaria. This information would have provided more insights on the possible transmission of malaria from pockets with indigenous cases within the townships. Fifth, the sample size was small and was not powered enough to assess the factors associated with delayed diagnosis.

The malaria cases were clustered along the foothills and increased during the rainy season in these townships. The higher rainfall, temperature and elevation are known to be associated with malaria [23]. However, in the current study, cases were clustered along the foothills compared to elevated areas. The clustering seen could be only due more number of cases in foothills which has relatively higher population density compared to elevated area. In contrary, the highly dense plain coastal areas had less number of cases. The potential reason for this could be the fact that the people living in foothills depend on forest in elevated area for their livelihood. The frequent visits to forest make the people vulnerable as they encounter several species of the malaria vectors present in forests [4]. Also, as these foothills have poor access to health services and transportation facilities, the diagnosis of malaria cases and response activities are delayed. This delay in treatment might have had increased the transmission of the malaria in foothills. Similar to findings in China and Bangladesh, the malaria cases showed seasonal pattern with high number of cases in rainy season [24, 25].

Our findings showed that about 88% of investigated cases were indigenous to the township. Previous study from Myanmar had reported a lower indigenous case (67%) rate [26]. This indicates the high endemicity of malaria within these townships. However, majority of the cases were imported from the other villages indicating low rate of transmission within the villages. Those who are dependent on forest for livelihood in the foothills of these townships are also seasonal agriculture workers moving across the villages in search of work. The movement of these agriculture workers is of concern as they can facilitate the transmission of disease to far-off villages. To achieve the elimination, the townships have to have zero indigenous case for three consecutive years. Thus, there is need for identifying and prioritizing the villages with high endemicity for focused effort to reduce transmission.

Delayed diagnosis of malaria cases remained critical issue in these townships. The diagnosis delay in this area was higher than that reported in other studies (40% to 79%) conducted elsewhere in Myanmar [11–13, 27]. The terrain and poor access to health services might be the potential reason for such delays. However, there is need for further exploring the reasons for delay to seek care once the symptom (fever) develops. Along with delay, about one in eight patient was treated not according to NTG. There is a lack of literature on compliance of malaria treatment prescription according to NTG in Myanmar. Although we did not explore the reason for this non-adherence to NTG, the anti-malaria drug stock out and lack of provider willingness to adhere to NTG might be the potential reasons. However, this warrants for a qualitative study to explore the reasons for deviation from NTG and take corrective actions.

There are several programmatic implications resulting from this study. First, the geotagging during CCI and generation of heat maps was feasible with in the programmatic setting. The maps generated provided insights on the heterogeneity of distribution of cases within the townships and across the seasons. Moving towards malaria elimination, the VBDC program can train the existing staff to collect the geocodes and generate township level malaria case distribution maps for informed decision making while prioritizing areas for control activities.

Second, about nine out of ten malaria cases were indigenous cases to the townships and were concentrated in the northern and southern borders of Toungup township. The programme needs to explore whether the concentrated vector control activities in these geospatially targeted areas would be effective in reducing number of malaria cases in the township. Third, majority of the cases acquired malaria within the townships, indicating potential risk of contracting malaria from the areas with high load of indigenous cases. The individuals travelling to areas with indigenous malaria cases, needs to be identified and actively screened for malaria especially during rainy season.

Fourth, the proportion who underwent CCI in the study setting was higher than that reported from study conducted in three low endemic regions of Myanmar [26]. The programme needs to explore the best practices that led to high CCI coverage and adopt those in other regions.

## Conclusions

In the targeted townships for malaria elimination, the high proportion of the cases was indigenous and clustered at the foothill areas during rainy season. The programme should strengthen case surveillance and healthcare services in the areas with aggregation of cases to eliminate the malaria in the township. As high majority of patients have delayed diagnosis, the reasons for delay has to be explored and corrective measures needs to be taken.

## Data Availability

The datasets used and/or analyzed during the current study are available from the corresponding author with a reasonable request.

## Abbreviations

API: Annual Parasite Incidence CCI Comprehensive Case Investigation GTS Global Technical Strategies LLIN Long Lasting Insecticide-treated Nets NMCP National Malaria Control Programme NSP National Strategic Plan NTG National Treatment Guideline PR Prevalence Ration RDT Rapid Diagnostic Test SEAR South-East Asia Region TSG Technical Support Group URC University of Research Co.,LLC VBDC Vector Borne Diseases Control Programme WHO World Health Organization
